# Migration and transformation of cadmium in rice - soil under different nitrogen sources in polymetallic sulfide mining areas

**DOI:** 10.1038/s41598-020-59409-1

**Published:** 2020-02-12

**Authors:** Xiaoxia Zhang, Xuexia Zhang, Shuji Lv, Lei Shi, Rongping Wang

**Affiliations:** 1 Guangdong Key Laboratory of Integrated Agro-environmental Pollution Control and Management, Guangdong Institute of Eco-environmental Science & Technology, Guangzhou, 510650 China; 2 National-Regional Joint Engineering Research Center for Soil Pollution Control and Remediation in South China, Guangzhou, 510650 China; 3 College of Agronomy, Hunan Agricultural University, Changsha, 410128 China; 4 School of Metallurgy and Environment, Central South University, Changsha, 410083 China

**Keywords:** Environmental sciences, Pollution remediation

## Abstract

We conducted pot experiments to assess the bioavailability of cadmium (Cd) in contaminated rhizosphere soil and accumulation in rice organs in response to nitrogen (N) supply ((NH_4_)_2_SO_4_, NH_4_NO_3_, NH_4_Cl). The results showed that the concentration of bioavailable Cd in rice rhizosphere soil was (NH_4_)_2_SO_4_ treatment > NH_4_Cl treatment > NH_4_NO_3_ treatment at the same level of N application and growth period; the Cd concentration in rice roots was (NH_4_)_2_SO_4_ treatment > NH_4_NO_3_ treatment > NH_4_Cl treatment; and the Cd concentration in rice straw was NH_4_NO_3_ treatment > NH_4_Cl. The Cd concentration in rice roots, straws, and seeds at the maturity stage was (NH_4_)_2_SO_4_ treatment > NH_4_Cl treatment. With the same N fertilizer, excessive N promoted Cd accumulation in rice at later growth stages. This suggested that sulfate (SO_4_^2−^) influenced Cd concentration in rice. NH_4_Cl application maintained a low Cd level in different rice organs with the same N level. This confirmed that NH_4_Cl is a safe N source for rice planting in polymetallic sulfide mining areas. The study concludes that appropriate NH_4_Cl levels for Cd-contaminated paddy soil with high-S-content could obtain rice grains with Cd concentrations below the food safety standards (0.2 or 0.4 mg·kg^−1^).

## Introduction

Mining has a tremendous impact on the geochemical environment of the mine and its surrounding ecosystems^1^. During the mining of polymetallic sulfide mines, metal sulfides release a large number of heavy-metal ions and acid wastewater^2^, which flow into natural waters, such as rivers, and result in the acidification and accumulation of heavy metals in paddy soils downstream of mining areas^3,4^ as well as in heavy metals in crops exceeding the standard in agricultural products, such as rice and maize^5–7^. Excessive SO_4_^2^^−^ retained in the soil leads to soil acidification and the formation of acid sulfate soil^8–10^. Plant nutrient regulation is essential to mitigate abiotic stress in sustainable agriculture. Mineral nutrients can significantly mitigate the accumulation of heavy metals in plants. In isolated studies, two essential mineral nutrients, nitrogen (N) and sulfur (S), have been reported to reduce the impact of Cd in plants and to improve overall plant growth, metabolism, and productivity under Cd exposure^11–13^. Some studies, however, have shown a positive correlation between S soures and Cd accumulation in plants^14,15^. Assimilation of SO_4_^2^^−^ and nitrate (NO_3_^−^) is involved in plant stress defense^11^. The S requirement and S metabolism of plants is closely related to N metabolism. The state of one element has a significant effect on the other^16,17^. Anjum *et al*.^18^ reported that assimilatory of SO_4_^2^^−^ and NO_3_^−^ reduction coordinated well in the mitigation of metal(loid) toxicity. Because of the optimization of a large N and S requirement, plants maintained healthy growth and, ultimately, a high yield under Cd stress^19,20^. Few reports, however, are available on the interactive effects of S and N sources in the mitigation of Cd toxicity^11^. Therefore, effort is needed to unravel the physiological mechanisms involved in the balance between Cd, S, N, and plant growth. We investigated the influence of different forms of N source on rice growth and Cd concentration in rice soil in polymetallic sulfide mining areas. We conducted pot experiments to assess the bioavailability of Cd in rhizosphere soil and examined its accumulation in different organs of *Choukoukoku* (Oryza sativa L.cv.)^21^ in response to various forms of N supply.

## Materials and Methods

### Soil

We collected the soil in this study from paddy fields polluted by heavy metals located downstream of the Dabaoshan polymetallic sulfide mining area in the northern Guangdong Province. Since the 1970s, this farmland has been using acid mine wastewater from Dabaoshan for sewage irrigation. This land has had a history of sewage irrigation for more than 40 years. The level of many heavy metals, including Cd, lead (Pb), and arsenic (As), in the local soils is between the risk screening values and the risk intervention values for soil contamination of agricultural land (GB 15618–2018). In principle, agronomic control and other safe utilization measures should be taken before planting edible agricultural products that do not meet the quality and safety standards for this kind of soil pollution. The tested soil was from 0- to 20-cm-deep topsoil of a tillage layer. After air-drying, we screened the soil through a 2-mm-pore nylon sieve, and then fully mixed the soil for the pot experiment. The basic physical and chemical properties of the tested soils are shown in Table 1.

**Table 1:** Basic physical and chemical properties of the tested soil.

	Risk screening value^a^	Risk intervention value^a^	Test value
pH	≤5.5	≤5.5	4.21
Cd/mg·kg^−1^	0.3	1.5	0.54
As/mg·kg^−1^	30	200	70.28
Cu/mg·kg^−1^	50		298.25
Zn/mg·kg^−1^	200		302.66
Cr/mg·kg^−1^	250	800	46.28
Pb/mg·kg^−1^	80	400	201.00
S/mg·kg^−1^			443.66
Bioavailable S/mg·kg^−1^			133.38

^a^Risk screening values and risk intervention values for soil contamination of agricultural land, soil environmental quality national standard of the People’s Republic of China, and risk control standard for soil contamination of agricultural land (GB 15618-2018).

### Potted experimental design

*Choukoukoku* (Oryza sativa L. cv.) is a rice variety with a high level of Cd accumulation. We treated the seeds of *Choukoukoku* using the following steps: (1) disinfected with 0.5% sodium hypochlorite for 20 minutes, (2) washed several times with deionized water, and (3) soaked for 24 hours. The treated seeds were cultured in damp gauze at room temperature. When they germinated to about 1 cm, we transferred the seeds to a pot containing quartz sand and cultured them in deionized water. When the seedlings grew three to four leaves, we selected seedlings with uniform growth for the soil culture experiment. We planted two plants in each pot.

We set up the N fertilizer varieties and their application levels in rice pots in nine treatments, as shown in Table 2. Each treatment had eight replicates.

**Table 2:** The test settings of N-level management.

Fertilizer	N (g N·kg^−1^soil)		
	0.1 g·kg^−1^	0.2 g·kg^−1^	0.4 g·kg^−1^
NH_4_Cl	7.6 g-A	15.2 g-B	30.4 g-C
(NH_4_)_2_SO_4_	9.4 g-D	18.8 g-E	37.6 g-F
NH_4_NO_3_	5.8 g-G	11.6 g-H	23.2 g-I
KH_2_PO_4_	17.6 g		

We prepared 72 pot devices two days before transplanting the rice seedlings. We filled 3.8 kg of soil in a plastic pot with a diameter of 20 cm and a height of 20 cm. At the same time, we weighed N and phosphorus fertilizers in proportion to the values shown in Table 2. We dissolved the fertilizer in 1000 mL of water and added it to the soil. To ensure the growth of seedlings, we added water to the water surface after transplanting, which was 2 cm higher than the soil interface. We kept the samples in a greenhouse and watered each pot every morning and evening.

### Collection and treatment of rice plants

We transplanted the rice seedlings on May 21, 2012. We collected rice plant samples at the tillering stage for analysis on July 2, 2012. When the rice plants ripened, they were harvested after three days of roasting. The ripening time of rice in each treatment was different, according to the ripening conditions, and the specific sampling dates of the ripening period were as follows: on August 23, 2012, the rice plant samples treated with A, B, D, E, G, H, and I in Table 2 were collected; on August 28, 2012, the rice plant samples treated with F in Table 2 were collected; on September 3, 2012, the rice plant samples treated with C in Table 2 were collected. Photos of the maturation stage were taken on August 23, 2012. They were provided as supplementary figures.

After collecting rice samples, we cut down the samples along the rice roots. We took out the rice roots along with the surrounding soil and collected the roots and the soil around the rhizospheric rice. We rinsed the rice roots, stems and leaves, and the grains with tap water and then rinsed them with deionized water. They were green-killed at 105 °C for 30 minutes, and then dried at 60 °C until the weight remained unchanged. Finally, the dried roots, stems and leaves, and grains were crushed and screened through a 60-mesh nylon sieve and then stored separately in polyethylene plastic bags. The rhizosphere soil samples were air-dried at room temperature and grinded with an agate mortar after removing animal and plant residues and other impurities. After screening through 10-mesh and 100-mesh nylon sieves, we stored the rhizosphere soil samples in separate polyethylene plastic bags.

### Sample testing and data analysis

We measured the soil pH value in deionized water at a soil-to-solution ratio of 1:2.5 (w: v). We determined the heavy-metal and S concentrations in the soil using an inductively coupled plasma optical emission spectrometer (ICP-OES, ICP-5000, Focused Photonics, Inc., Zhejiang, China), following HNO_3_-HClO_4_-HF digestion. The detection limits of the ICP-OES measurements were 0.5 µg·L^−1^ for Cd; 1 µg·L^−1^ for Cr; 0.5 µg·L^−1^ for Cu; 0.5 µg·L^−1^ for As; 0.5 µg·L^−1^ for Pb; 0.2 µg·L^−1^ for Zn; and 0.2 mg·L^−1^ for S. We determined the bioavailable concentrations of Cd in the soil using ICP-OES, following 1:2 suspensions of soil and 0.005 mol·L^−1^ DTPA − 0.01 mol·L^−1^ CaCl_2_ − 0.1 mol·L^−1^ TEA mixed solution leaching at 180 rotations per minute (rpm) for 2 hours. We also determined the bioavailable concentrations of S in the soil using ICP-OES, following 1:5 suspensions of soil and 0.008 mol·L^−1^ Ca(H_2_PO_4_)_2_ − 2 mol·L^−1^ CH_3_COOH mixed solution leaching at 180 rpm for 1 hour. The organs samples of rice were digested by a HNO_3_-HClO_4_ (3:1, v: v) mixed acid.

In the sample pretreatment process, the acid reagents were of high purity (G.R.). In the process of sample detection, we added a standard solution to every 20 samples with an internal standard method quality control. The recoveries of Cd in soil and plant samples were higher than 95%, and the relative standard deviation was more than 10%.

All of the data were statistically analyzed using Microsoft Excel 2007 and DPS 7.05 software and were expressed as means ± standard deviation. Treatment means were compared using least significant difference at p < 0.05, and the graphics were drawn using Origin 8.0.

## Results and Discussion

### Bioavailable Cd and pH of rice rhizosphere soil under different N fertilizer treatment conditions

A large number of studies have found that different forms of N fertilizer application can cause different changes in soil pH, thus affecting the bioavailability of heavy metals in rhizosphere soil^22–24^.

**Figure 1:**
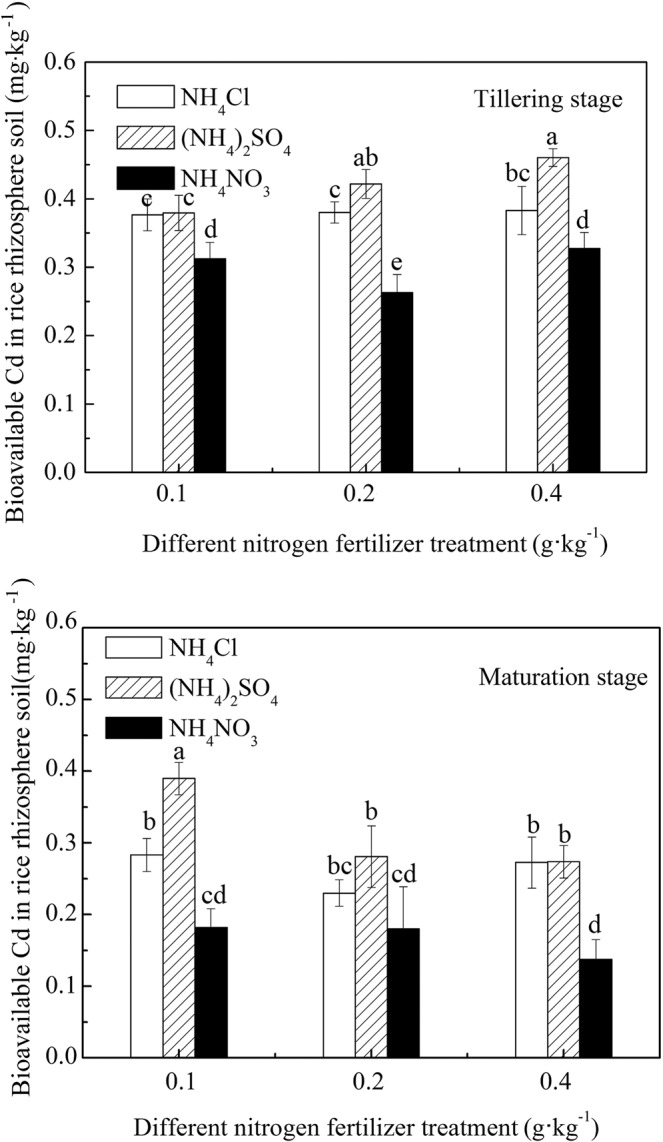
Concentration of bioavailable Cd in the rhizosphere soil under different N fertilizer treatment conditions.

The effects of different N fertilizer treatments on bioavailable Cd content in rice rhizosphere soil at tillering and maturing stages are shown in Fig. 1. The bioavailable Cd content in rice rhizosphere soil at the tillering and maturing stages under the three N fertilizer treatment conditions were as follows: (NH_4_)_2_SO_4_ > NH_4_Cl > NH_4_NO_3_. This content indicated that the supply of S significantly increased the bioavailability of Cd in the soil. This also indirectly indicated that the effect of NH_4_^+^-N on the bioavailability of Cd in the soil was more significant than that of NO_3_^−^-N. This result is consistent with Eriksson’s^24^ finding that NH_4_^+^-N significantly increased Cd activity in soil. With an increase in N fertilizer application, the change trend of bioavailable Cd was different. At the tillering stage, when NH_4_Cl and (NH_4_)_2_SO_4_ were applied, the change trend of available Cd content in rice rhizosphere soil was the same as that of the N level, which increased with an increase in the N level; however, the increase of the (NH_4_)_2_SO_4_ treatment was more obvious. The content of bioavailable Cd under (NH_4_)_2_SO_4_ treatments increased the most rapidly. With an increase of N application, the content of bioavailable Cd increased from 0.379 mg·kg^−1^ to 0.460 mg·kg^−1^, increasing by 21.37%. The increase in the application NH_4_Cl fertilizer did not significantly increase Cd bioavailability, increasing only slightly from 0.376 mg·kg^−1^ to 0.383 mg·kg^−1^. The NH_4_NO_3_ application had the smallest effect on the increase of bioavailable Cd content in rice rhizosphere soil. The bioavailable Cd content in rice rhizosphere soil under 0.1 g·kg^−1^, 0.2 g·kg^−1^, and 0.4 g·kg^−1^ of NH_4_NO_3_ treatment conditions was only 82.41%, 62.35%, and 71.15% under (NH_4_)_2_SO_4_ application, respectively. The content of bioavailable Cd first decreased and then increased with an increase in the N level. At the maturity stage, the bioavailable Cd content in the rhizosphere soil of rice decreased with an increase in the N application, from 0.411 mg·kg^−1^ and 0.182 mg·kg^−1^ to 0.274 mg·kg^−1^ and 0.137 mg·kg^−1^, and decreased by 33.33% and 24.72%, respectively. Under NH_4_Cl fertilizer treatments, the bioavailable Cd content in rhizosphere soil first decreased and then increased, and it reached the lowest level at the treatment level of 0.2 g·kg^−1^. Compared with bioavailable Cd content at the tillering stage and maturity stage, the effect of NH_4_^+^-N application to improve soil Cd bioavailability was more significant than that of NO_3_^−^-N, and the supply of S significantly increased the soil Cd bioavailability.

**Figure 2:**
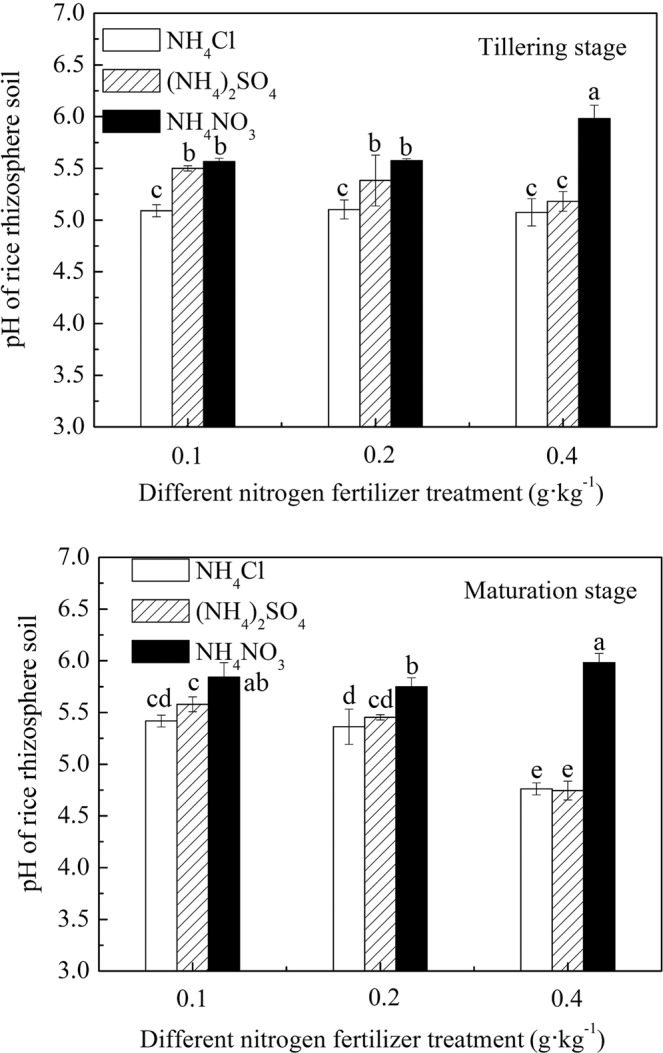
pH of rice rhizosphere soil under different N fertilizer treatment conditions.

The effect of different N fertilizer treatments on the pH of rice rhizosphere soil is shown in Fig. 2. The pH of rice rhizosphere soil was different under conditions of the same growth period, the same N application rate, and different N fertilizer varieties. The results showed that the pH of rice rhizosphere soil under (NH_4_)_2_SO_4_ and NH_4_Cl treatments was lower than under NH_4_NO_3_ treatments. When plants absorb NH_4_^+^-N and NO_3_^−^-N, their roots secrete different ions. When they absorb NH_4_^+^-N, it causes H^+^ secretion, which results in acidification of the rice rhizosphere soil. Conversely, when they absorb NO_3_^−^-N, they secrete OH^−^, which results in alkalization of the rhizosphere soil. In this experiment, these three N fertilizers introduced the same cations into the soil, and with the same N level, NH_4_^+^ contained in (NH_4_)_2_SO_4_ and NH_4_Cl was greater than NH_4_NO_3_. Therefore, the acidification of the first two N fertilizers was greater than that of the latter, and the bioavailable Cd content in the corresponding soil was higher (as shown in Fig. 1).

In addition, previous studies have shown that the anions introduced by the three N fertilizers also have some influence on the adsorption of heavy metals in soil, because Cl^−^ can form relatively stable complexes with Cd in solution (such as CdCl^+^, CdC1_2_^0^, CdC1_3_^−^, CdCl_4_^2−^, and so on), which causes Cd to migrate from a solid to the soil solution and to improve the solubility of Cd^25^.

Consensus has not been reached on the effect of heavy-metal uptake by plants under S^11–15^. Our previous studies have shown, however, that when rice is planted in polluted soils of polymetallic sulfide mining areas, both total S and bioavailable S were enriched in rice rhizosphere soil, and the S content in the rhizosphere soil was positively correlated with Cd accumulation in rice^26^. This result indicated that an increase in the SO_4_^2−^ content also may increase Cd bioavailability. In addition, SO_4_^2−^ also competed for the adsorption sites of Cd ions on the soil surface, which increased the release of Cd in soil solution^27^. The effects of different N fertilizers on soil pH and Cd bioavailability under flooding conditions have been studied by Jiaka *et al*.^28^. The results showed that the ability order of N fertilizer to reduce soil pH was NH_4_Cl > (NH_4_)_2_SO_4_ > NH_4_NO_3_, and the ability order to increase soil Cd bioavailability under N fertilizer was NH_4_Cl > (NH_4_)_2_SO_4_ > NH_4_NO_3_^28^. The results of this experiment showed that the ability to increase soil Cd bioavailability was (NH_4_)_2_SO_4_ > NH_4_Cl. These differences may have resulted from the different physicochemical properties of the paddy soil planted in the two experiments. The former soil used was from a rice paddy soil developed from gray alluvial deposits in Sichuan, and its pH was 6.57, which was a neutral paddy soil. The soil used in this experiment was paddy soil that had been irrigated by sewage from the polymetallic sulfide mining area for many years. The acid mine drainage not only caused the decreasing soil pH (pH = 4.21), but also caused the increasing S content in the soil. The total S content in the soil was 443.66 mg·kg^−1^, which was 1.58 times higher than the average S content in Guangdong Province. The bioavailable S content was 133.38 mg·kg^−1^, which was 6.2 times higher than the average bioavailable S content in Guangdong Province, with 30% of the total S content, which was higher than 10% of the average S content in natural soil. This finding indicated that the soil contained a large number of highly mobile sulfate ions. Our previous studies showed that both total and bioavailable S tended to be enriched in rice rhizosphere soil, which also resulted in the existence of Cd in rhizosphere soil in the form of higher bioavailability^26^.

### Cd concentration in rice roots under different N fertilizer treatment conditions

**Figure 3:**
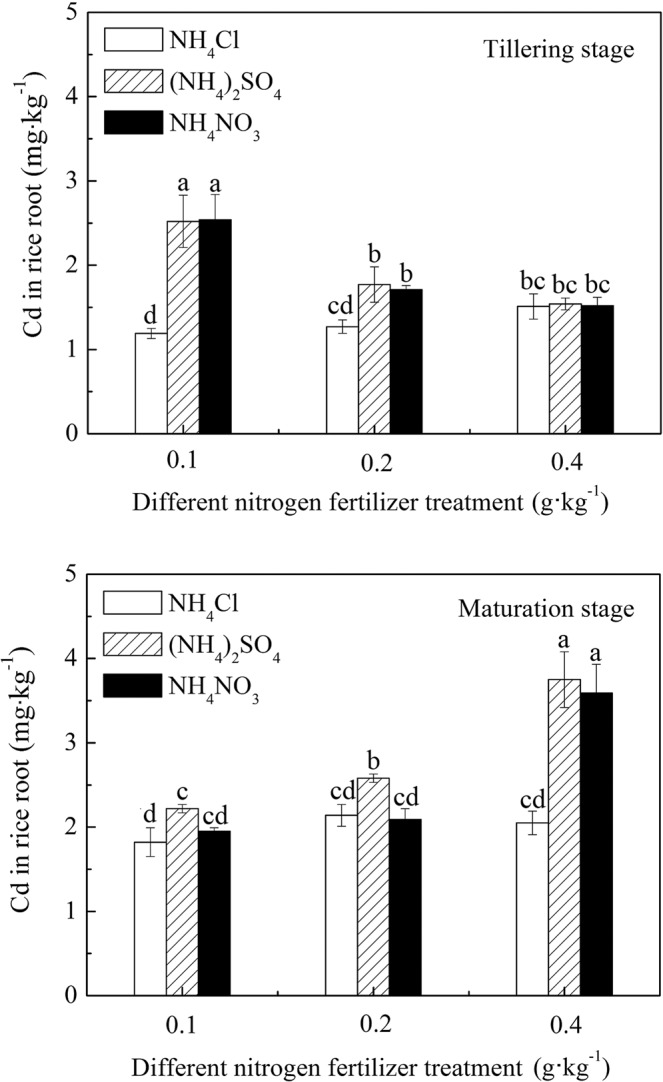
Cd concentration in rice roots under different N fertilizer treatment conditions.

Figure 3 shows that the Cd concentration in rice roots was different during the same growth periods under the same amount of different N sources. The Cd concentration in rice roots was greatest under (NH_4_)_2_SO_4_ application, followed by NH_4_NO_3_, and NH_4_Cl was the lowest. The effects of the three N fertilizer treatments on Cd concentration in rice roots were as follows: (NH_4_)_2_SO_4_ > NH_4_NO_3_ > NH_4_Cl. The effects of Cd concentration in roots at different growth stages of rice under different N fertilizer treatments were different. At the tillering stage, the Cd concentration in the rice roots increased along with the increasing level of NH_4_Cl, which increased from 1.19 mg·kg^−1^ to 1.51 mg·kg^−1^. The Cd content in the rice roots did not differ much under the same N level of (NH_4_)_2_SO_4_ and NH_4_NO_3_, but it decreased with an increase in the N level. This result indicated that increasing the amount of these two N fertilizers would inhibit the absorption of Cd in rice roots during the early growth period. Except for the treatment of the 0.1 g·kg^−1^ N application level under (NH_4_)_2_SO_4_ and NH_4_NO_3_ treatments, the Cd content in rice roots increased more significantly at the ripening stage than at the tillering stage. This result indicated that the Cd concentration in the rice roots increased with rice growing, but the effects of different N fertilizer application on Cd concentration in the rice roots were different. With an increase in the NH_4_Cl application treatments, the Cd content in the rice roots remained basically unchanged. With an increase in the (NH_4_)_2_SO_4_ and NH_4_NO_3_ treatment conditions, the Cd content in the rice roots increased significantly. These results showed that Cd absorption in the rice roots increased under the (NH_4_)_2_SO_4_ and NH_4_NO_3_ N fertilizer treatment at the later growing stage.

A comparison of Fig. 1 with Fig. 3 shows that the bioavailable Cd content in the paddy soils treated with the three N fertilizers was (NH_4_)_2_SO_4_ treatment > NH_4_Cl treatment > NH_4_NO_3 _treatment, whereas the Cd content in the rice roots was (NH_4_)_2_SO_4_ > NH_4_NO_3_ > NH_4_Cl. This comparison also shows that different forms of N fertilizer have different effects on the Cd content in paddy soils and rice roots. Xie *et al*.^22^ studied the effects of different N forms on the uptake and accumulation of Cd and zinc by hyperaccumulating plant *Cymbidium nigrum*. According to their results, NH_4_^+^-N treatment decreased the pH of the rhizosphere soil more than NO_3_^−^-N treatment, whereas NH_4_^+^-N treatment promoted the accumulation of Cd in *Cymbidium nigrum* more than NH_4_^+^-N treatment. In addition, Hassan *et al*.^29^ studied the effects of N forms on Cd toxicity in rice. Their results showed that the Cd content in rice roots and stalks was Ca(NO_3_)_2_ treatment > NH_4_NO_3_ treatment > (NH_4_)_2_SO_4_ treatment, which aligned with the conclusion that the Cd content in plant roots and stems was NO_3_^−^-N treatment > NH_4_^+^-N treatment. The comparative results of NH_4_Cl and NH_4_NO_3_ treatments in this experiment were the same as those in the noted studies. NH_4_^+^ and H^+^ competed with Cd ions for the root adsorption sites. In contrast, NH_4_^+^-N treatment could make the root secrete more malic acid chelating with Cd than with NO_3_^−^-N treatment^29,30^.

In this study, the bioavailable Cd content in the rice rhizosphere soil and in rice roots was the highest in the treatment group of (NH_4_)_2_SO_4_. The main reason for this difference was that the experimental soil was paddy soil that had been polluted by acid mine wastewater and contained excessive SO_4_^2−^, which promoted the absorption of Cd by the rice roots^26,31^.

### Cd concentration in rice stems and leaves under different N fertilizer treatment conditions

**Figure 4:**
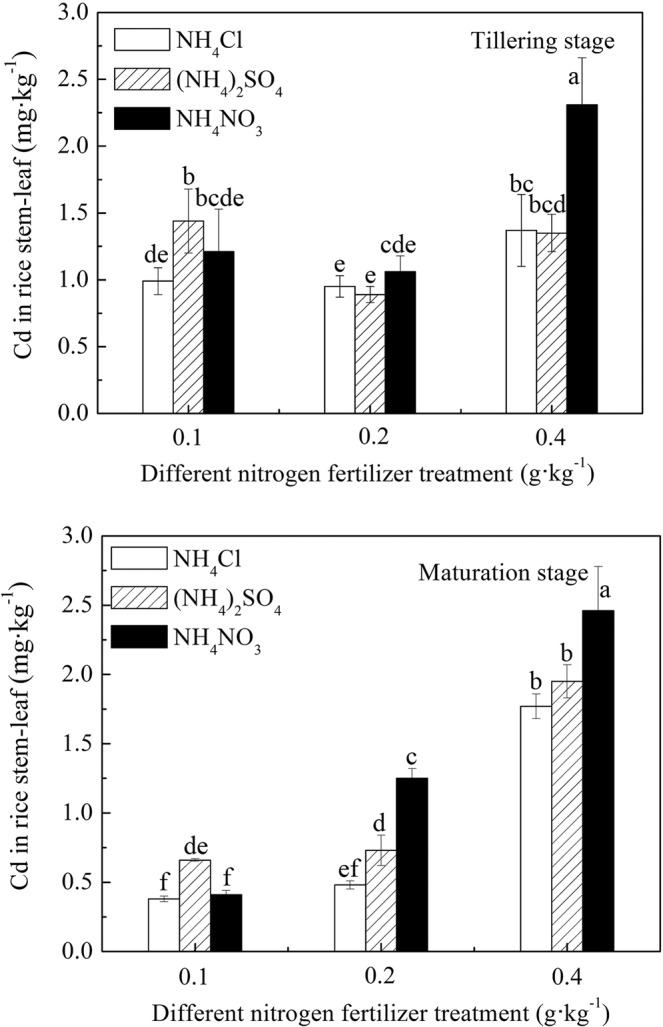
Cd concentration in rice stems and leaves under different N fertilizer treatment conditions.

Figure 4 shows the effects of different levels of N fertilizer treatments on Cd concentration in rice stems and leaves at the tillering and maturity stages. The Cd concentration in rice stems and leaves under the same growth period and N application rate was as follows: NH_4_NO_3_ > NH_4_Cl. The results were the same as that for the rice roots. When the N level gradually increased, the application of NH_4_NO_3_ fertilizer was more conducive to the concentration and accumulation of Cd by rice stems and leaves. This result was the same as that found by Jönsson *et al*.^32^. The application of NH_4_NO_3_ was more beneficial to Cd accumulation in plant stems than that of NH_4_Cl. The benefit may be related to the transport of NO_3_^−^ in rice stems and leaves. Other studies have shown that the transport of NO_3_^−^ to plant stems requires the participation of cations to achieve a charge balance, which will promote the transfer of Cd to the stem^32^. Plants usually absorb NH_4_^+^-N and cause H^+^ secretion, which results in soil acidification around the plant rhizosphere. When plants absorb NO_3_^−^-N and cause OH^−^ secretion, it results in rhizosphere soil alkalization. Therefore, it is generally believed that NO_3_^−^-N is a more suitable application than NH_4_^+^-N in acid soil. This study, however, showed that in an acid-heavy metal-contaminated soil remediation project, fertilization could not be selected only by adjusting the soil pH value. The selection of N fertilizer should be considered on the basis of the effect of different forms of N fertilizer on the migration of heavy metals to plants.

Comparing Figs. 3 and 4, the Cd concentration in rice stems and leaves was different from that in the rice root treated with (NH_4_)_2_SO_4_. With the 0.1 g·kg^−1^ N treatment level, the Cd concentration in the rice stems and leaves remained as follows: (NH_4_)_2_SO_4_ treatment > NH_4_NO_3_ treatment > NH_4_Cl treatment, which followed the same trend as that in the rice root. The Cd content in the rice stems and leaves following the other two treatments was lower than that with the NH_4_NO_3_ treatments. This result suggested that application of (NH_4_)_2_SO_4_ reduced Cd transport from the rice root to the stems and leaves. Gao studied the effect of SO_4_^2−^ on Cd accumulation in rice seedlings^31^. She noted that SO_4_^2−^ enhanced the retention ability of Cd in the rice root tissues and reduced the transfer of Cd to the shoots. When the content of SO_4_^2−^ increased to a certain extent and exceeded the retention ability of Cd in roots, the Cd began to transfer to the shoots. This indicated that a certain mutual restriction existed between SO_4_^2−^ and N applications on Cd absorption. The soil in this experiment was polluted by acid mine wastewater, and its S content, especially bioavailable S, was high, which may have hindered the transfer of Cd to shoots.

Figure 4 shows that the Cd concentration in rice stems and leaves increased with an increase in the N level at the maturity stage under the same N fertilizer variety treatment. This finding indicated that the application of N fertilizer could promote the Cd concentration in rice stems and leaves at the later stage of rice growing. Under the same growth period and N treatment level (except 0.1 g·kg^−1^), the Cd concentration in rice stems and leaves was the highest with NH_4_NO_3_ treatments. Figure 4 also shows that the Cd concentration in rice stems and leaves at the tillering stage was more serious than that at the ripening stage. The content of bioavailable Cd in the soil during this period may have been higher; conversely, rice grows vigorously during the vegetative growth stage and passively absorbs more Cd.

### Cd concentration in brown rice under different N fertilizer treatment conditions

**Figure 5:**
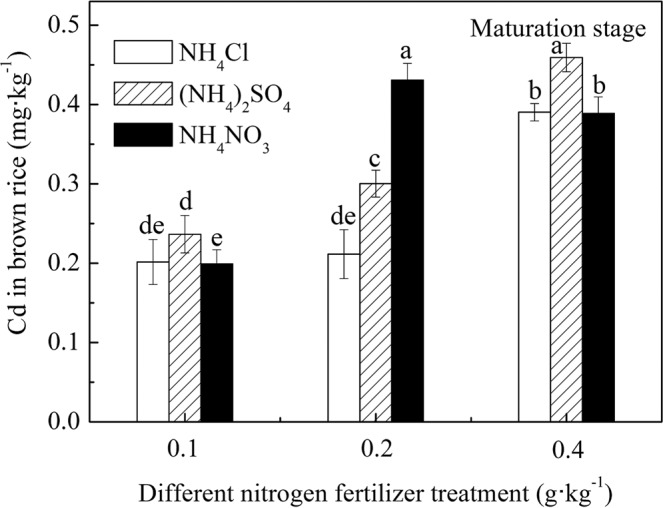
Cd concentration in brown rice under different N fertilizer treatment conditions.

The Cd concentration of brown rice under different N fertilizer treatment conditions is shown in Fig. 5. Figure 5 shows that under the N application conditions selected in this experiment, the Cd concentration in brown rice exceeded the 0.2 mg·kg^−1^ standard (GB 2762–2017)^33^, but the exceeding standard was different for the different N fertilizer varieties and N levels. Other than the 0.4 g·kg^−1^ N application level of the NH_4_NO_3_ treatment, the Cd content in brown rice increased gradually with an increase in the N application level. The Cd concentration in brown rice treated with NH_4_Cl increased from 0.202 mg·kg^−1^ to 0.390 mg·kg^−1^, but the highest Cd content in brown rice treated with NH_4_NO_3_ reached 0.431 mg·kg^−1^ at the 0.2 g·kg^−1^ N application level, and then it decreased to 0.390 mg·kg^−1^. Therefore, the effects of NH_4_NO_3_ and NH_4_Cl treatments on the Cd concentration in rice seed did not show the same regularity as that of rice straw. At the 0.4 g·kg^−1^ N application level, the amount of N fertilizer was high, which resulted in the late maturity of rice treated with NH_4_Cl and (NH_4_)_2_SO_4_ (more than five days later than the 0.1 g·kg^−1^ N application level and the 0.2 g·kg^−1^ N application level). In general, the Cd concentration in brown rice was the lowest when treated with NH_4_Cl.

The Cd content in brown rice treated with NH_4_Cl and (NH_4_)_2_SO_4_ increased gradually with an increase in the N application level. The Cd concentration of brown rice treated with NH_4_Cl increased from 0.202 mg·kg^−1^ to 0.390 mg·kg^−1^, and the Cd concentration in brown rice treated with (NH_4_)_2_SO_4_ increased from 0.236 mg·kg^−1^ to 0.459 mg·kg^−1^. The Cd concentration of brown rice was (NH_4_)_2_SO_4_ treatments > NH_4_Cl treatments, which was the same as that of rice roots and straws at the ripening stage. Compared with NH_4_NO_3_ treatments, the Cd concentration change trend in brown rice at the 0.1 g·kg^−1^ N application level and the 0.2 g·kg^−1^ N application level was the same as that in rice straw. At the 0.4 g·kg^−1^ N application level, the Cd content in brown rice was the highest when treated with (NH_4_)_2_SO_4_. This result may be due to the excessive SO_4_^2−^ content, which exceeded the ability of the root to retain Cd and promoted the transfer of Cd to the shoot^31^.

No consensus has been reached on the effect of N fertilizer applications on Cd accumulation in rice. Eriksson^24^ showed that the order of Cd uptake by plants was (NH_4_)_2_SO_4_ > NH_4_NO_3_, Zeng *et al*.^34^ showed that Cd uptake by rice seedlings treated with the three N fertilizers was NH_4_NO_3_ > NH_4_Cl > (NH_4_)_2_SO_4_. Jiaka *et al*.^28^ showed that different forms of N fertilizer promoted Cd uptake and that the order of Cd content in rice straw was (NH_4_)_2_SO_4_ > NH_4_Cl > NH_4_NO_3_, whereas Cd content in rice grain was NH_4_Cl > NH_4_NO_3_ > (NH_4_)_2_SO_4_. Notably, S, as a necessary nutrient for plants as well as an important component in soil, has some restrictive effects with NH_4_^+^-N and NO_3_^−^-N. In paddy soils with high S content, the application of N fertilizer should consider the effect of SO_4_^2−^, and NH_4_Cl fertilizer seems more suitable for high-S-content soil.

As for the effect of N sources on rice growth, the photos taken of the first mature samples (collected on August 23) show that the biomass of rice treated with NH_4_Cl and (NH_4_)_2_SO_4_ was higher than that treated with NH_4_NO_3_. The results of Jiaka *et al*.^28^ and Jalloh *et al*.^35^ showed that NH_4_^+^-N treatments had significantly higher grain yields, which was consistent with our results. The picture taken on August 23 and the information in Table 3 show that under the 0.4 g·kg^−1^ N application level, the excessive N source enabled the rice treated with NH_4_Cl and (NH_4_)_2_SO_4_ to have extended growth and delayed maturity (about 5–21 days later than the 0.1 g·kg^−1^ and 0.2 g·kg^−1^ N application levels). Because N is the main component of chlorophyll, the greener the plant, the higher the N concentration it has^36^. The green color of the rice was NH_4_Cl treatment > (NH_4_)_2_SO_4_ treatment > NH_4_NO_3_ treatment. Therefore, the N concentration in rice was NH_4_Cl treatment > (NH_4_)_2_SO_4_ treatment > NH_4_NO_3_ treatment, but the Cd concentration in rice was (NH_4_)_2_SO_4_ treatment > NH_4_Cl treatment ≈ NH_4_NO_3_ treatment. Jalloh *et al*.^35^ also showed that NH_4_^+^-N treatments had significant higher N accumulation in plant tissues than NO_3_^−^-N treatments. The research results from Yang^37^, however, showed that rice plantlets treated with NH_4_^+^ had a higher growth ability with higher tillers, dry weight, photosynthetic rate, transpiration rate, and total N content by 49.1%, 9.6%, 106.5%, 9.6%, and 48.3%, respectively, when compared with NO_3_^−^-treated plantlets under Cd stress. The NO_3_^−^-treated rice plantlet had the strongest Cd absorption, which was 2.79 times higher than NH_4_^+^ treated in rice roots. Figures 4 and 5 show that rice treated with 0.2 g·kg^−1^ and 0.4 g·kg^−1^ NO_3_^−^-N had a higher Cd concentration but was less green in color, and thus its N concentration was lower. Therefore, the N concentration in aboveground rice was high, and the Cd concentration was correspondingly high under the same form of N source. For rice treated with different forms of the same N level, this phenomenon was not obvious. Figures 4 and 5 show that the Cd concentration increased significantly from 0.2 gN·kg^−1^ to 0.4 gN·kg^−1^. The probable reason for this increase was that excessive NH_4_^+^ enhanced Cd translocation by inhibiting root heavy-metal adenosinetriphosphatase gene expression in rice and that excessive NO_3_^−^ enhanced Cd uptake by up-regulating the expression of iron-regulated metal transporter in rice^37,38^.

**Table 3:** Days of rice growth period.

Fertilizer	N (g N·kg^−1^soil)		
	0.1 g·kg^−1^	0.2 g·kg^−1^	0.4 g·kg^−1^
NH_4_Cl	109 d-A	109 d-B	(120–131) d-C
(NH_4_)_2_SO_4_	109 d-D	109 d-E	(114–123) d-F
NH_4_NO_3_	109 d-G	109 d-H	109 d-I

In addition, generally the bioavailable Cd content in rice rhizosphere soil is high, which indicates that the Cd content can be absorbed by rice also is high. This study, however, showed that although the bioavailable Cd content in soil was positively correlated with the Cd absorption by rice roots, it was not strongly correlated with the Cd content in rice straw and rice seed. Therefore, it remains questionable which chemical extraction method should be used to characterize the bioavailability of Cd in soil.

Rice Cd standards are different in different countries. The current World Health Organization Cd standard is 0.4 mg·kg^−1^, and this standard currently is used in Japan and Taiwan. The European Union and China have a stricter standard of 0.2 mg·kg^−1^. Figure 5 shows that all of the Cd concentrations in the brown rice treated with NH_4_Cl did not exceed 0.4 mg·kg^−1^. Therefore, the Cd concentration in rice can be kept at a low level by applying NH_4_Cl source in high-S-content paddy soil. For field production where the experimental soil samples were collected, the Cd concentration of brown rice was between 2.35 and 3.50 mg·kg^−1^ under farmers’ habitual way of applying fertilizer. Considering that the results of pot incubation and field production are different, the Cd concentration for aboveground pot incubation usually is lower than the concentration for field production^26,39^. Therefore, future work is required to optimize the NH_4_Cl supply concentration under field conditions to obtain rice grains with Cd concentration below the food safety standards of 0.2 or 0.4 mg·kg^−1^. If necessary, agricultural measures, such as low-Cd-uptake rice varieties and water management^26^, can be used.

## Conclusions

We conducted pot experiments to study the effects of different N fertilizer varieties and levels on bioavailable Cd in rice rhizosphere soil and the Cd concentration in different organs of rice at the tillering and maturing stages. The effect of NH_4_^+^-N on improving the bioavailability of Cd in soil was more significant than that of NO_3_^−^-N; and the supply of S significantly increased the bioavailability of Cd in soil. The bioavailable Cd content in rice rhizosphere soil and in rice roots was the highest when treated with (NH_4_)_2_SO_4_. However, S and N applications had some mutual restrictions on the Cd concentration. At a certain level of N, application of (NH_4_)_2_SO_4_ reduced Cd transport from rice roots to shoots. When it exceeded that level, application of (NH_4_)_2_SO_4_ promoted the transfer of Cd from rice roots to shoots. The Cd concentration with NH_4_Cl treatment was relatively lower at different growth stages than with (NH_4_)_2_SO_4_ and NH_4_NO_3_ treatment. Thus, this study suggests that NH_4_Cl would be a good N source to reduce Cd phytoextraction for paddy soil with a high level of S content and likely would obtain rice grains with Cd concentration below the food safety standards of 0.2 or 0.4 mg·kg^−1^.

## Supplementary information

Supplementary figures.
