# Supplementary material for: Migration and transformation of cadmium in rice - soil under different nitrogen sources in polymetallic sulfide mining areas
Source: Sci Rep. 2020 Feb 12;10:2418. doi: 10.1038/s41598-020-59409-1 (PMC7016120; doi:10.1038/s41598-020-59409-1)

Migration and transformation of cadmium in rice-soil under different nitrogen sources in polymetallic sulfide mining areas

Xiaoxia Zhang[1,2*], Xuexia Zhang[1], Shuji Lv[3], Lei Shi[4] & Rongping Wang[1,2*]

[1]Guangdong Key Laboratory of Integrated Agro-environmental Pollution Control and Management, Guangdong Institute of Eco-environmental Science & Technology, Guangzhou 510650, China. [2]National-Regional Joint Engineering Research Center for Soil Pollution Control and Remediation in South China, Guangzhou 510650, China. [3]College of Agronomy, Hunan Agricultural University, Changsha 410128, China. [4]School of Metallurgy and Environment, Central South University, Changsha 410083, China. [*]email:zhangxiaoxia.20088@163.com; rpwang@soil.gd.cn

Supplementary figures. Photos of maturation stage taken on August 23, 2012.

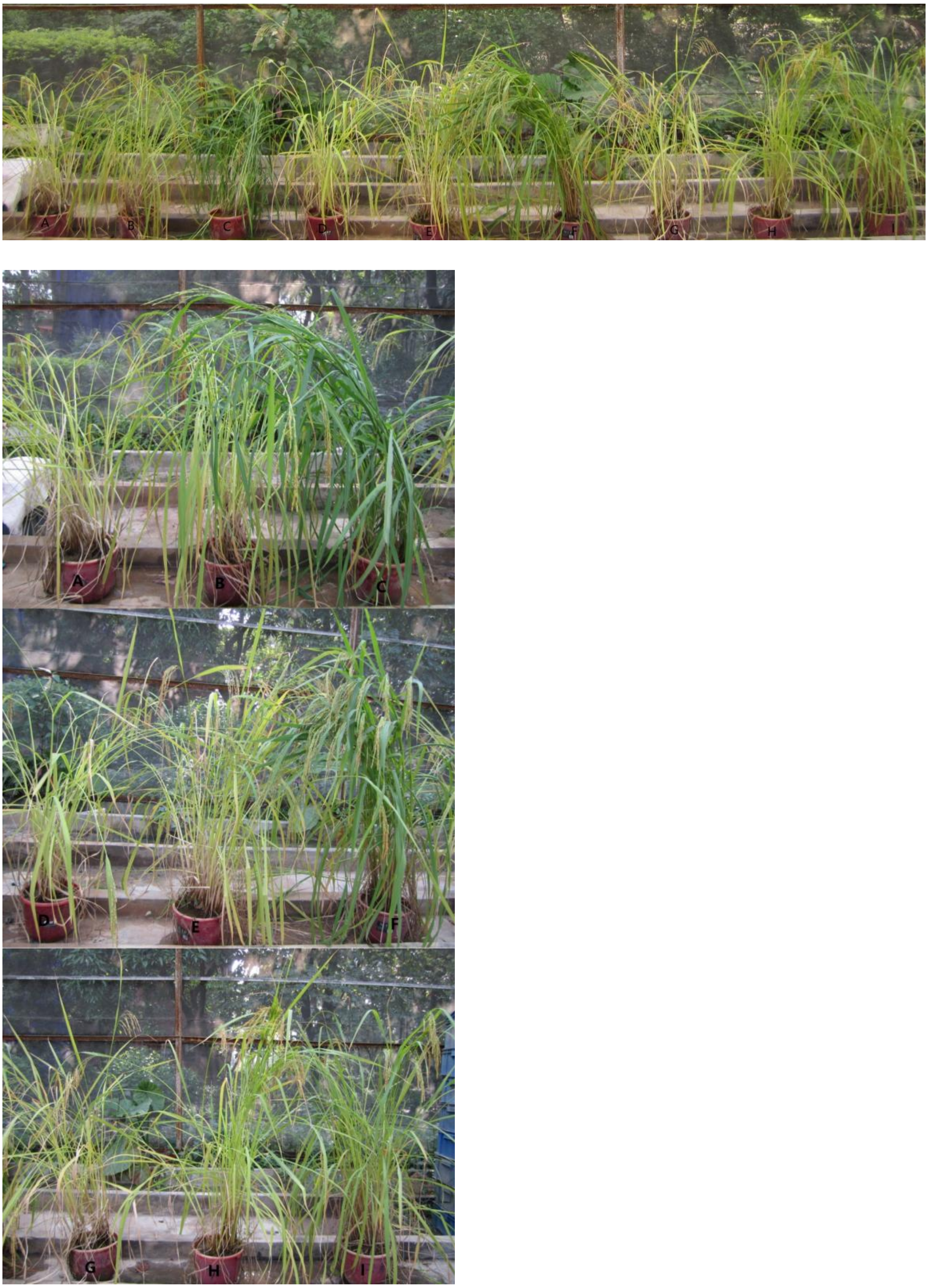

Supplement: Supplementary file 1 — Supplementary figures. [file 41598_2020_59409_MOESM1_ESM.pdf]
